# Supplementary material for: Factors associated with the very high caesarean section rate in urban areas of Vietnam
Source: PLoS One. 2022 Aug 29;17(8):e0273847. doi: 10.1371/journal.pone.0273847 (PMC9423634; doi:10.1371/journal.pone.0273847)
Supplement: Attachment — Submitted filename: Response to Reviewers.docx [file pone.0273847.s002.docx]

**Dear Editorial Board Member**

**PLOS ONE**

Re: *Factors associated with the very high caesarean section rate in urban areas of Vietnam*

Thank you very much for the opportunity to revise this manuscript. We have addressed the comments in the point-by-point response.

We hope that our responses are satisfactory to you.

Yours sincerely,

Hoang Thi Nam Giang

On behalf of all authors

**Reviewer’s comments**

***Comment****: Thanks for the opportunity to review this manuscript. It was very well-written and the limitations are clearly described. It drives interesting evidences on the uprising of cesarean section in Vietnan, and I think the results presented would be similar in other countries with similar development. I only suggest that maternal and infant characteristics that were considered for adjustment are not described, neither in methods nor in table 2. Consider including it.*

**Response**: Thanks for your comments. We described maternal and infant characteristics that were considered for adjustment in methods and in Table 2 as follow:

Page 6, line 128 and 132: “Maternal and infant characteristics were included in multivariable logistic regression are maternal age, educational level, place of residence, average family income, parity, complication during pregnancy and at birth, mode of delivery, pre-pregnancy body mass index (BMI), gestational weight gain (kg), infant’s sex, and infant’s birth weight”

Thank you for your comments.
